# Exosomal lncRNAs as molecular switches driving macrophage-tumor co-evolution in breast cancer

**DOI:** 10.1007/s10238-026-02199-z

**Published:** 2026-05-31

**Authors:** Mahsa Javadian, Tahereh zeinali, Morteza Rajabi, Zahra Mehrasa, Mahsa Boogari, Faezeh Ramezani, Saeed Samaeinasab, Zahra Mahmoudi, Hossein Hakimi, Rozhina Sabzehban, Khadijeh Najafi, Kiana Esmaeili, Zohreh Khaksarhaghani, Safa Tahmasebi, Ramazan Rezaei

**Affiliations:** 1 Immunology Research Center, Shiraz University of Medical Science, Shiraz, Iran; 2 Department of Immunology, school of Medicine, Shiraz University of Medical Science, Shiraz, Iran; 3 Student Research Committee, Shiraz University of Medical Sciences, Shiraz, Iran; 4 Gastrointestinal and Liver Diseases Research Center, Guilan University of Medical Sciences, Rasht, Iran; 5 Primary Immunodeficiency Diseases Network (PIDNet), Universal Scientific Education and Research Network (USERN), Tehran, Iran; 6 Pediatric Cell & Gene Therapy Research Center, Gene, Cell, and Tissue Research institute, Children’s Medical Center, Tehran University of Medical Sciences, Tehran, Iran; 7 Cancer Research Center, Tehran University of Medical Sciences, Tehran, Iran; 8 Department of Biology, Faculty of Basic Sciences, Central Tehran branch, Islamic Azad University, Tehran, Iran; 9 Department of Medical Genetics, School of Medicine, Iran University of Medical Science, Tehran, Iran; 10 Departmemt of Medical Genetics, School of Medicine, Tehran University of Medical Science, Tehran, Iran; 11 Division of Medical Biotechnology, Department of Medical Laboratory Sciences, School of Paramedical Sciences, Shiraz University of Medical Sciences, Shiraz, Iran; 12 Blood Transfusion Research Center, High Institute for Research and Education in Transfusion Medicine, Tehran, Iran; 13 Immunology Board for Transplantation and Cell-Based Therapeutics (ImmunoTACT), Universal Scientific Education and Research Network (USERN), Chicago, IL USA; 14 Immunology Research Center, Kurdistan University of Medical Sciences, Sanandaj, Iran; 15 Student Research committee, drug applied research center, Tabriz University of Medical sciences, Tabriz, Iran; 16 Department of Oncology and Metabolism, School of Medicine, The University of Sheffield, Sheffield, UK; 17 School of Medicine, Shahrekord University of Medical Sciences, Shahrekord, Iran; 18 Department of Immunology, School of Medicine, Shahid Beheshti University of Medical Sciences, Tehran, Iran; 19 Department of Immunology, Medical Faculty, Shahed University, Tehran, Iran; 20 Department of Immunology, Faculty of Medicine, Shahed University, Tehran, Iran

**Keywords:** Breast Neoplasms, Extracellular Vesicles, Long Noncoding RNA, Tumor-associated macrophages Tumor Microenvironment

## Abstract

Breast cancer progression is increasingly recognized as a dynamic process shaped by reciprocal communication between malignant cells and the tumor microenvironment (TME). Among immune-cell populations, tumor-associated macrophages (TAMs) are major regulators of immune suppression, metabolic adaptation, angiogenesis, and therapeutic resistance. Emerging evidence indicates that extracellular vesicle (EV)-mediated transfer of long non-coding RNAs (lncRNAs) constitutes an important mechanism underlying tumor–immune communication in breast cancer. In this review, we summarize mechanistically characterized studies examining how exosomal lncRNAs regulate bidirectional signaling between breast cancer cells and macrophages and contribute to tumor progression, immune remodeling, and resistance-associated phenotypes. Tumor-derived exosomal lncRNAs modulate macrophage signaling through pathways associated with signal transducer and activator of transcription 3 (STAT3), transforming growth factor beta (TGF-β), Hippo/Yes-associated protein (YAP), hypoxia-responsive signaling, and autophagy-related remodeling, thereby promoting immunoregulatory and tumor-supportive macrophage phenotypes. Conversely, macrophage-derived exosomal lncRNAs, including hypoxia-inducible factor-1 alpha (HIF-1α)-stabilizing long non-coding RNA (HISLA), reinforce glycolytic adaptation, epithelial–mesenchymal transition, epigenetic remodeling, metastatic plasticity, and resistance to therapy in recipient tumor cells. Exosomal lncRNA signaling additionally influences γδ T cells, endothelial cells, and stromal compartments, supporting broader multicellular regulation within the TME. Collectively, current evidence supports exosomal lncRNAs as biologically important mediators of tumor–immune adaptation in breast cancer. We further discuss the translational potential of circulating exosomal lncRNAs as minimally invasive biomarkers and evaluate therapeutic strategies targeting EV biogenesis, vesicle trafficking, and oncogenic lncRNA cargo molecules. Finally, we highlight current limitations involving EV heterogeneity, lncRNA stoichiometry, and incomplete in vivo validation that remain critical barriers to clinical translation.

## Introduction

Breast cancer remains a major global health burden, with incidence and mortality driven disproportionately by metastatic relapse and therapy-refractory disease. Contemporary frameworks increasingly recognize that these outcomes are not determined solely by tumor cell-intrinsic alterations, but by adaptive interactions within the tumor microenvironment (TME), where immune and stromal compartments can either constrain or potentiate malignant progression [1–3]. Among immune populations in the breast TME, tumor-associated macrophages (TAMs) are frequently abundant and clinically relevant, yet their biology is better captured by a spectrum of activation states than by strict binary labels. The M1/M2 macrophages shorthand can remain a useful conceptual tool, but macrophage activation is context-defined and shaped by local cytokines, metabolites, and tissue cues, which collectively regulate programs linked to tumor growth, matrix remodeling, angiogenesis, and immune suppression [4–7].

A major reason TAMs exert durable effects is their participation in continuous bidirectional communication with malignant cells. Beyond soluble cytokines and chemokines, extracellular vesicles (EVs) provide a protected route for transferring complex regulatory cargo between cells, enabling signaling outputs that are sustained and layered rather than transient. Current consensus guidance also recommends “EV” as the umbrella term in view of practical limits in proving vesicle biogenesis pathways in many experimental settings [8]. Within EVs, exosomes are commonly discussed as a functional subpopulation generated through intraluminal budding in multivesicular bodies and released upon fusion with the plasma membrane. Their cargo can include proteins, lipids, and regulatory RNAs that reprogram recipient cells at transcriptional and post-transcriptional levels, providing a mechanism for long-range tuning of immune tone, metabolic state, and invasive behavior within the TME [8, 9].

Long non-coding RNAs (lncRNAs) have emerged as particularly versatile EV cargo because they can regulate gene expression by acting as competing endogenous RNAs (ceRNAs), scaffolding regulatory complexes, or shaping chromatin-associated programs. LncRNA have emerged as particularly versatile EV cargo because they can regulate gene expression by acting as competing endogenous RNAs (ceRNAs), scaffolding regulatory complexes, or shaping chromatin-associated programs. Importantly, lncRNA loading into EVs is not necessarily stochastic; rather, it may be influenced by RNA-binding proteins, sequence-dependent motifs, and RNA modifications that bias selective cargo sorting, supporting the possibility that EV-associated lncRNAs participate in biologically organized intercellular communication processes [10, 11].

Despite growing interest in exosomal lncRNA biology, several conceptual and technical limitations remain. Many proposed signaling pathways are derived from overexpression or knockdown systems that may not fully reflect endogenous EV cargo abundance or physiological stoichiometry in vivo. In addition, EV heterogeneity, variability in isolation methodologies, incomplete characterization of recipient-cell uptake mechanisms, and differences in vesicle purification protocols continue to complicate mechanistic interpretation and cross-study reproducibility [8, 12, 13]. Consequently, functional assignment of exosomal lncRNA signaling pathways should remain restricted to experimentally validated biological contexts supported by robust recipient-cell and pathway-specific evidence.

This review therefore examines the hypothesis that exosomal lncRNAs contribute to reciprocal communication between breast cancer cells and TAMs, thereby influencing epithelial-mesenchymal transition (EMT), angiogenesis, metabolic adaptation, immune suppression, and therapeutic resistance within the TME. Rather than proposing a universally conserved signaling network, we focus on mechanistically characterized pathways that collectively support the concept of dynamic tumor–immune communication mediated by extracellular vesicle-associated lncRNAs. Accordingly, we first introduce macrophage-derived EVs as context-dependent communication mediators within the breast TME and summarize EV biogenesis and lncRNA sorting mechanisms most relevant to pathway regulation. We next synthesize mechanistically characterized studies in which tumor-derived exosomal lncRNAs modulate macrophage signaling programs and macrophage-derived exosomal lncRNAs reinforce tumor-cell metabolic adaptation, hypoxia-responsive signaling, invasive plasticity, and epigenetic remodeling. As an illustrative example of reciprocal metabolic communication, we highlight the macrophage-to-tumor transfer of hypoxia-inducible factor-1 alpha (HIF-1α)-stabilizing long non-coding RNA (HISLA), which promotes glycolysis and chemoresistance through lactate-associated feedback interactions. Finally, we discuss translational implications while emphasizing current limitations involving EV heterogeneity, signaling stoichiometry, and reproducibility across experimental systems.

## Macrophage exosomes in the breast TME

The breast TME is composed of malignant epithelial cells embedded within a dynamic stromal and immune network that includes endothelial cells, cancer-associated fibroblasts, adipocytes, and infiltrating leukocytes. Among immune subsets, TAMs are consistently enriched in aggressive breast tumors and are strongly associated with angiogenesis, metastasis, and reduced overall survival. Clinical correlative analyses and experimental models indicate that high TAM density predicts poor prognosis, particularly in triple-negative and high-grade disease [1, 7, 14].

TAMs arise largely from circulating monocytes that are recruited by chemokines such as CCL2 and colony-stimulating factor 1 (CSF1), and they differentiate under the influence of tumor-derived cytokines, hypoxia, and metabolic stress. Although the M1/M2 macrophages dichotomy is reductive, it remains useful in illustrating how macrophages can adopt inflammatory, tumor-restrictive states or immunoregulatory, tissue-remodeling phenotypes. In breast cancer, TAMs frequently display features associated with alternative activation, including increased signal transducer and activator of transcription 3 (STAT3) signaling, enhanced secretion of interleukin 10 (IL-10) and transforming growth factor beta (TGF-β), and support for angiogenesis and extracellular matrix remodeling. These states are governed by integrated pathways such as Janus kinase and STAT, nuclear factor kappa B (NF-κB), phosphoinositide 3-kinase and protein kinase B (PI3K/AKT), and HIF-1α, which couple inflammatory cues to metabolic adaptation [5, 7, 15, 16].

EVs provide a mechanism by which these macrophage states can be imposed on neighboring compartments. Consensus guidelines define EVs as lipid bilayer-enclosed particles released by cells that carry proteins, lipids, and nucleic acids; exosomes are commonly described as a small EV subtype originating from multivesicular endosomes, typically ranging from 30 to 150 nanometers in diameter [8, 17]. In the breast TME, both tumor cells and macrophages release EVs that can be internalized by neighboring or distant cells, allowing transfer of regulatory cargo that modulates gene expression and signaling in recipient cells [18]. Tumor-derived EVs have been shown to skew macrophages toward tumor-supportive phenotypes, but macrophage-derived EVs are equally important in shaping tumor behavior. TAM exosomes can transfer proteins, lipids, and non-coding RNAs that enhance cancer cell proliferation, invasion, and drug resistance. The macrophage EV cargo has been implicated in reinforcing hypoxia signaling, metabolic adaptation, and EMT-associated transcriptional programs in recipient tumor cells. Such effects are context-dependent and reflect the activation state of the macrophage donor cell [19, 20].

The influence of macrophage EVs extends beyond direct tumor-cell modulation. TAM-derived vesicles can affect endothelial cells and stromal components, contributing to angiogenesis and matrix remodeling. Pro-angiogenic signaling in endothelial cells, including activation of PI3K/AKT and mitogen-activated protein kinase (MAPK) cascades, has been linked to EV-mediated transfer of bioactive cargo from myeloid cells. These interactions illustrate how macrophage exosomes act as multipronged regulators of TME architecture rather than isolated immune modulators [1, 6, 21, 22]. Macrophage EVs also intersect with adaptive immunity. TAM-derived vesicles can carry ligands or regulatory RNAs that suppress cytotoxic T-cell responses or favor regulatory T-cell expansion, thereby contributing to immune tolerance. Conversely, experimental reprogramming of macrophages toward inflammatory states can alter EV cargo composition and enhance anti-tumor immune activation, underscoring the plasticity of macrophage-derived vesicle function [2, 7, 23].

The macrophage-derived exosomes represent a central communication hub in the breast TME. Their cargo composition reflects macrophage activation state and environmental context, and their functional reach extends from immune modulation to metabolic reprogramming and vascular remodeling. This dual capacity-to reinforce tumor-promoting phenotypes or, under defined conditions, support anti-tumor immunity-establishes macrophage exosomes as pivotal regulators of intercellular signaling. The following sections therefore focus specifically on lncRNAs within these vesicles and how they drive reciprocal tumor-macrophage co-evolution.

## Exosomal lncRNAs: biogenesis and functional principles

LncRNAs are transcripts longer than 200 nucleotides with limited or absent protein-coding potential that regulate gene expression at transcriptional, post-transcriptional, and epigenetic levels. They are transcribed primarily by RNA polymerase II, frequently spliced and polyadenylated, and can arise from intergenic, intergenic, or antisense genomic regions. In cancer biology, lncRNAs have been implicated in proliferation, EMT, stemness, metabolic adaptation, and therapy resistance. Their functional diversity derives from their ability to interact with DNA, RNA, and proteins, thereby modulating chromatin state, transcription factor activity, and microRNA (miRNA) availability [18, 24, 25].

A distinct and increasingly important dimension of lncRNA biology is their selective incorporation into EVs, particularly small EVs commonly referred to as exosomes. Exosomes originate within multivesicular bodies through inward budding of the endosomal membrane and are released upon fusion with the plasma membrane. This process can be regulated by endosomal sorting complexes required for transport (ESCRT) components, as well as ESCRT-independent mechanisms involving lipids and tetraspanins. Rab GTPases such as Rab27a and Rab27b contribute to vesicle docking and secretion, underscoring the active, regulated nature of exosome biogenesis [26–28].

Selective loading of lncRNAs into exosomes is not a passive reflection of cellular abundance. RNA-binding proteins (RBPs) recognize specific sequence motifs or structural elements and guide RNAs toward vesicular compartments. Heterogeneous nuclear ribonucleoprotein A2/B1 (hnRNPA2B1) has been shown to bind defined RNA motifs and regulate RNA sorting into exosomes, while Y-box binding protein 1 (YBX1) participates in RNA packaging into small EVs. These findings support a model in which donor-cell signaling states influence exosomal cargo composition through regulated RBP-RNA interactions [11, 17, 26, 29].

**Fig. 1:**
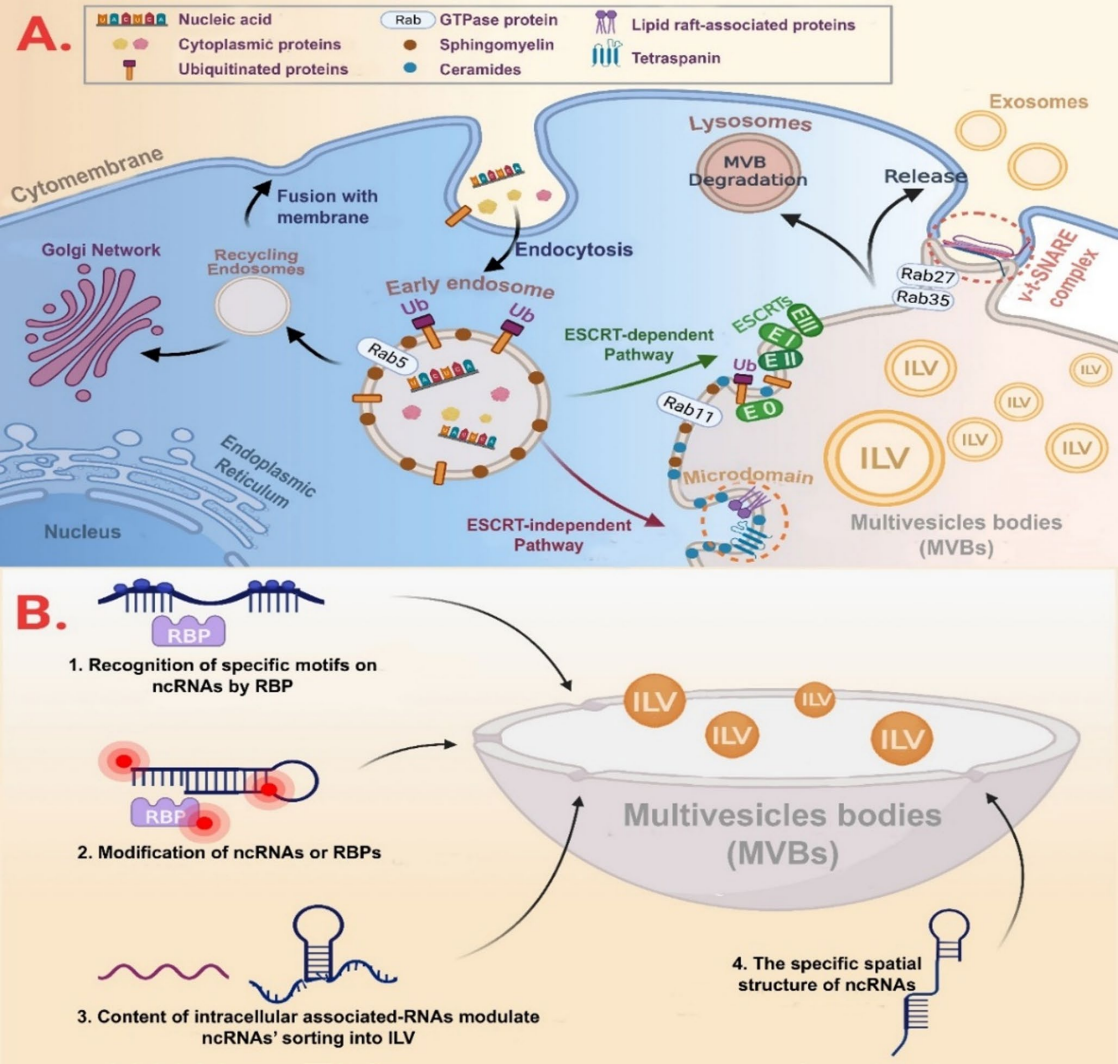
Schematic overview of extracellular vesicle biogenesis and selective non-coding RNA sorting mechanisms. Plasma membrane budding and endocytic trafficking generate early endosomes that mature into MVBs. ILVs are formed within MVBs through ESCRT-dependent and ESCRT-independent mechanisms involving lipids, tetraspanins, and RNA-binding proteins. MVBs subsequently fuse either with lysosomes for cargo degradation or with the plasma membrane to release ILVs as extracellular vesicles commonly referred to as exosomes. Selective incorporation of non-coding RNAs into exosomes is regulated by RNA-binding proteins and epitranscriptomic modifications, including m6A; ESCRT: Endosomal sorting complexes required for transport; EV: Extracellular vesicle; ILV: Intraluminal vesicle; m6A: N6-methyladenosine; MVB: Multivesicular body

Epitranscriptomic modification adds an additional layer of control. N6-methyladenosine (m6A), a prevalent internal RNA modification, influences RNA stability, localization, and protein binding. Emerging evidence indicates that m6A marks can promote selective enrichment of RNAs in exosomes through recognition by specific reader proteins, linking cellular stress or oncogenic signaling to vesicular RNA output. Although much of this work has been performed outside breast cancer specifically, the principle that RNA modification affects exosomal sorting is broadly supported and mechanistically plausible in tumor-immune communication contexts [25, 29, 30]. Once secreted, exosomes interact with recipient cells through endocytosis, phagocytosis, receptor-mediated uptake, or membrane fusion. After internalization, vesicular membranes are disrupted within endosomal compartments, releasing RNA cargo into the cytoplasm. Depending on sequence features and interacting partners, lncRNAs may remain cytoplasmic-acting as ceRNAs that sequester miRNAs or translocate to the nucleus to recruit chromatin-modifying complexes and reshape transcriptional programs. These mechanisms enable lncRNAs to influence signaling pathways involving transcription factors, metabolic regulators, inflammatory mediators, and epigenetic modifiers in recipient cells (Fig. 1) [11, 17].

An important unresolved issue in extracellular vesicle biology concerns the quantitative stoichiometry of lncRNA cargo delivery and whether sufficient transcript abundance is consistently achieved in recipient cells to support proposed ceRNA-mediated signaling effects. In addition, recipient-cell uptake efficiency, vesicle biodistribution, intracellular RNA stability, and competition with endogenous RNA networks may substantially influence the biological impact of exosomal lncRNA transfer [8, 12, 13, 31]. Therefore, mechanistic interpretation of exosomal lncRNA pathways should consider both vesicular cargo abundance and recipient-cell uptake efficiency when evaluating biological relevance, particularly in studies relying predominantly on overexpression or in vitro co-culture systems.

Functionally, several principles appear particularly relevant for tumor-macrophage communication in breast cancer. First, exosomal lncRNAs may alter microRNA availability and thereby modulate post-transcriptional repression networks linked to inflammatory signaling, metabolic adaptation, or hypoxia-responsive pathways. Second, certain lncRNAs can interact with chromatin-associated regulators, including polycomb repressive complex 2 (PRC2), potentially contributing to durable transcriptional remodeling in recipient cells. Third, exosomal lncRNAs may integrate stress-responsive pathways such as hypoxia, inflammatory signaling, or metabolic stress into broader adaptive responses within the tumor TME. Collectively, these mechanisms support the concept that extracellular vesicle-mediated lncRNA transfer contributes to dynamic intercellular communication between tumor cells and immune-cell populations rather than functioning solely as passive biomarker release.

In the context of breast cancer, these biogenetic and functional properties create the structural basis for reciprocal macrophage tumor signaling. Tumor-derived exosomal lncRNAs can reprogram macrophage signaling networks, while macrophage-derived lncRNAs can reinforce tumor-cell metabolic and invasive programs. Thus, exosomal lncRNA biogenesis, selective cargo sorting, and recipient-cell uptake represent important mechanistic processes that may shape reciprocal signaling interactions between breast cancer cells and macrophages within the TME. Understanding how these pathways influence immune regulation, metabolic adaptation, and therapeutic resistance remains essential for evaluating the translational relevance of exosomal lncRNA biology in breast cancer.

## Tumor-derived exosomal lncRNAs reprogramming macrophage signaling in the breast TME

**Fig. 2:**
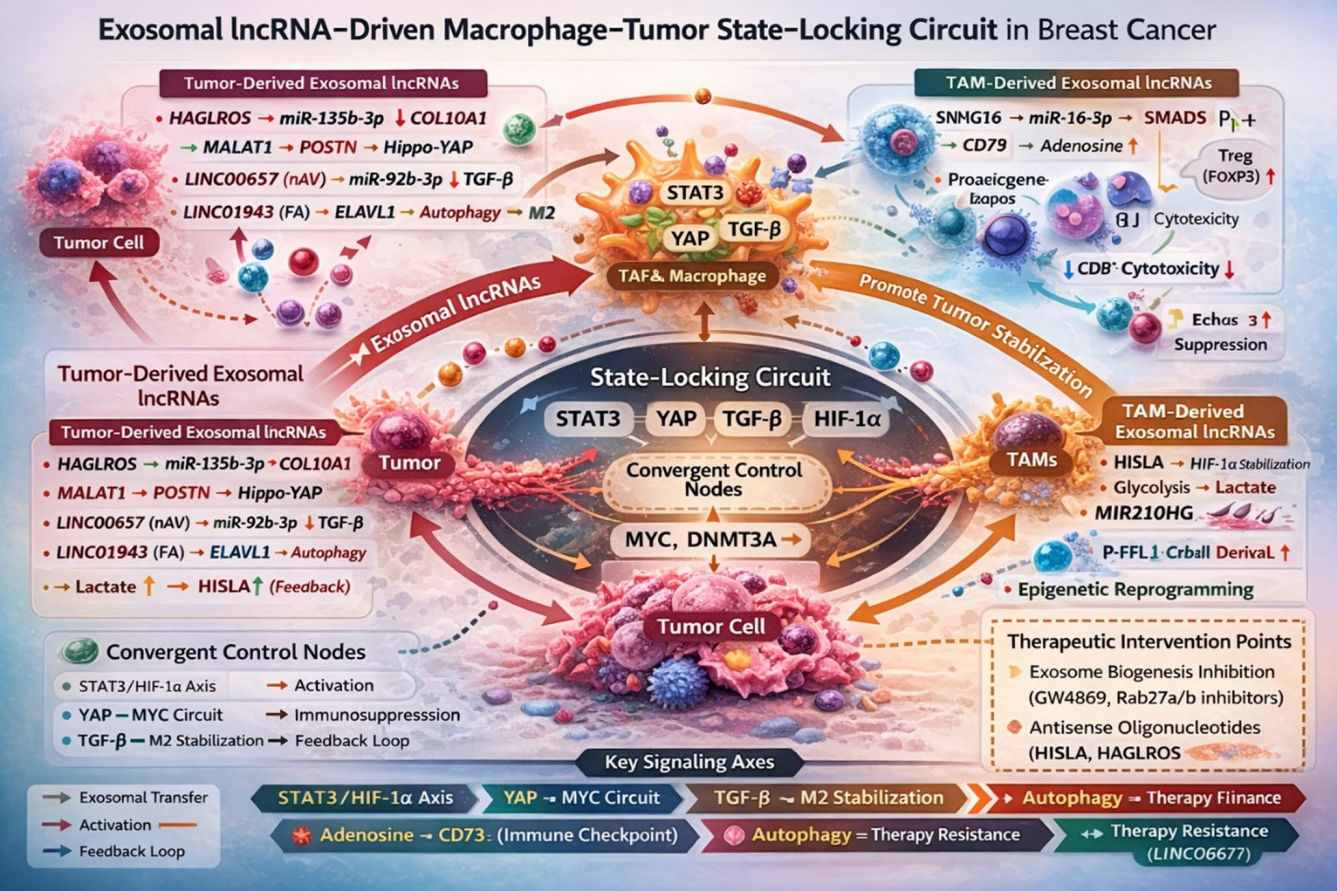
Exosomal lncRNA-driven macrophage–tumor communication network in breast cancer. Tumor-derived exosomal lncRNAs, including HAGLROS, MALAT1, LINC00657, and LINC01943, modulate macrophage phenotypes through distinct signaling pathways involving STAT3-associated polarization, Hippo/YAP signaling, TGF-β activation, and ELAVL1/autophagy-related regulation. In parallel, TAM-derived exosomal lncRNAs, including HISLA, MIR210HG, and LINC00470, reinforce tumor-cell metabolic adaptation, hypoxia-responsive signaling, epigenetic remodeling, and invasive plasticity through HIF-1α-associated pathways, MYC/DNMT3A activation, and epithelial–mesenchymal transition-associated programs. Tumor-derived SNHG16 additionally contributes to immune suppression by promoting CD73-dependent adenosine signaling in γδ T cells. Collectively, these bidirectional interactions establish a dynamic immunometabolic communication network that supports angiogenesis, immune tolerance, therapeutic resistance, and tumor progression; ASO: Antisense oligonucleotide, CD73: Ecto-5′-nucleotidase, ceRNA: Competing endogenous RNA, COL10A1: Collagen type X alpha 1, DNMT3A: DNA methyltransferase 3 alpha, ELAVL1: ELAV-like RNA-binding protein 1, EMT: Epithelial–mesenchymal transition, EV: Extracellular vesicle, HIF-1α: Hypoxia-inducible factor 1 alpha, HISLA: HIF-1α-stabilizing long noncoding RNA, lncRNA: Long non-coding RNA, m6A: N6-methyladenosine, MALAT1: Metastasis-associated lung adenocarcinoma transcript 1, miR: MicroRNA, MIR210HG: MIR210 host gene, MYC: MYC proto-oncogene, PHD2: Prolyl hydroxylase domain-containing protein 2, POSTN: Periostin, RASSF7: Ras association domain family member 7, SMAD5: SMAD family member 5, SNHG16: Small nucleolar RNA host gene 16, STAT3: Signal transducer and activator of transcription 3, TAM: Tumor-associated macrophage, TGF-β: Transforming growth factor beta, TME: Tumor microenvironment, YAP: Yes-associated protein

Breast cancer cells can remodel the immune architecture of the TME, by releasing exosomes that deliver long non coding RNAs, lncRNAs, into TAMs [32]. In the mechanistically resolved studies compiled in Table 1; Fig. 2, exosome-transferred lncRNAs modulate macrophage signaling programs associated with immunoregulatory, tissue-remodeling, and tumor-supportive phenotypes within the breast cancer microenvironment, with experimentally supported mechanisms involving partially overlapping but biologically distinct signaling pathways associated with STAT3, Hippo/YAP, TGF-β, hypoxia-responsive signaling, and epigenetic regulation [29, 32].

**Table 1:** Functional roles of tumor-derived exosomal lncRNAs in breast cancer

Tumor exosomal lncRNA	Type of breast cancer	Macrophage phenotype/source	Experimental model	Primary molecular target(s)	Dominant signaling pathway(s) / functional axis	Functional impact on TME	References
HAGLROS	BC (tissues + plasma exosomes)	TAM/M2 polarization (macrophages treated with BC exosomes)	In vitro + in vivo	miR-135b-3p → COL10A1; TAM M2 polarization with increased p-STAT3 signaling	HAGLROS/miR-135b-3p/COL10A1 with STAT3-associated M2 polarization	Promotes immunoregulatory macrophage polarization with M2-associated features; increases proliferation, migration, EMT, and angiogenesis	[32]
MALAT1 (TNBC EV-shuttled)	TNBC	M2 polarization (macrophages)	In vitro + in vivo	POSTN (regulated downstream of EV-MALAT1 in macrophages)	MALAT1 → POSTN → Hippo/YAP signaling → M2 polarization program	M2 polarization; promotes TNBC proliferation/invasion/metastasis and immunosuppressive remodeling	[25, 33, 34]
LINC00657 (m6A-dependent packaging)	BC	M2 polarization (macrophages)	In vitro + in vivo	miR-92b-3p; TGF-β signaling activation in macrophages; m6A modification/packaging (METTL3-related) in tumor cells	Exosomal LINC00657 → miR-92b-3p inhibition → TGF-β pathway activation → M2 polarization → tumor reinforcement loop	M2 activation; M2 macrophages feedback to enhance malignant phenotypes of BC cells	[18, 29, 35]
LINC01943 (RT context)	BC (radiotherapy resistance context)	M2 polarization (macrophages)	In vitro + in vivo	ELAVL1-mediated autophagy pathway regulation (study-defined)	RT-induced tumor exosomal LINC01943 → macrophage M2 polarization → RT resistance support	Promotes RT resistance via macrophage reprogramming	[25, 36]
SNHG16 (tumor-derived exosomes)	BC	Immune recipient = Vδ1 T cells (not macrophages)	Cell-based immune assays	miR-16-5p → SMAD5; ↑ CD73	SNHG16/miR-16-5p/SMAD5 → TGF-β1/SMAD5 activation → CD73 upregulation (adenosine immunosuppression)	Immunosuppression via CD73↑ (adenosine axis), favors immune escape	[37]
SNHG4	TNBC	Not tested (no macrophage recipient experiment reported)	In vitro + in vivo	XPO5 (reported target)	Exosomal SNHG4 transfer → XPO5 modulation → TNBC proliferation/migration (tumor-centric; macrophage recipient not tested)	Promotes TNBC progression (macrophage effect not primary endpoint)	[38]
MALAT1 (general BC exosomal MALAT1)	BC	Not assessed	In vitro + clinical samples	Multiple context-dependent targets	Exosome-mediated MALAT1 transfer → increased BC cell proliferation	Growth/proliferation in recipient tumor cells	[25, 33, 34]
lnc-MTRNR2L12-3 (hypoxia-induced BC exosomes)	Hypoxic BC context	Not assessed (angiogenesis focus)	Endothelial assays + in vivo angiogenesis	Src/FAK signaling activation in endothelial cells (HUVECs)	Hypoxic BC exosomal lnc-MTRNR2L12-3 → Src/FAK pathway activation → angiogenesis	Enhanced angiogenesis / vascular remodeling	[39]
NEAT1 (tumor–tumor EV signaling)	BC (paclitaxel-resistant)	Not tested (tumor recipient)	In vitro + xenograft	miR-133b axis (reported); CXCL12 not consistently validated as primary downstream effector in exosomal context	Exosomal NEAT1 → miR-133b repression → paclitaxel resistance and enhanced migration	Resistance dissemination among tumor cells; enhanced motility	[40]
HOTAIR (circulating exosomes; biomarker-linked)	BC (clinical correlation)	Not assessed	Patient blood exosomes + correlations	Clinicopathologic correlation (circulating exosomal HOTAIR detected in patient blood; associated with disease features)	Context-dependent epigenetic/ceRNA roles (mechanism not proven in this biomarker study)	Biomarker potential; macrophage recipient function not established	[41, 42]

BC: Breast cancer; CD73: Ecto-5′-nucleotidase; ceRNA: Competing endogenous RNA; COL10A1: Collagen type X alpha 1; CXCL12: C-X-C motif chemokine ligand 12;

ELAVL1: ELAV-like RNA-binding protein 1; EMT: Epithelial–mesenchymal transition; EV: Extracellular vesicle; FAK: Focal adhesion kinase; HIF-1α: Hypoxia-inducible factor 1 alpha; HOTAIR: HOX transcript antisense intergenic RNA; HUVEC: Human umbilical vein endothelial cell; lncRNA: Long non-coding RNA; lnc-MTRNR2L12-3: Long non-coding mitochondrial 2-like 12 − 3; m6A: N6-methyladenosine; MALAT1: Metastasis-associated lung adenocarcinoma transcript 1; METTL3: Methyltransferase-like 3; miR: MicroRNA; MIR210HG: MIR210 host gene; MYC: MYC proto-oncogene; NEAT1: Nuclear enriched abundant transcript 1; p-STAT3: Phosphorylated signal transducer and activator of transcription 3; POSTN: Periostin; RASSF7: Ras association domain family member 7; RT: Radiotherapy; SMAD5: SMAD family member 5; SNHG16: Small nucleolar RNA host gene 16; Src: Src proto-oncogene tyrosine-protein kinase; STAT3: Signal transducer and activator of transcription 3; TAM: Tumor-associated macrophage; TGF-β: Transforming growth factor beta; TME: Tumor microenvironment; TNBC: Triple-negative breast cancer; XPO5: Exportin-5; YAP: Yes-associated protein; YBX1: Y-box binding protein 1

### HAGLROS/miR-135b-3p/COL10A1/STAT3 axis in TAM M2 polarization

Breast cancer tissues and circulating plasma-derived exosomes exhibit elevated expression of lncRNA HAGLROS, which has emerged as an important regulator of macrophage polarization within the TME. Following exosomal transfer into macrophages, HAGLROS functions as a ceRNA by sequestering microRNA-135b-3p (miR-135b-3p), thereby relieving repression of collagen type X alpha 1 (COL10A1). Increased COL10A1 expression is associated with enhanced STAT3 phosphorylation, a signaling event strongly linked to immunoregulatory macrophage activation and M2-associated phenotypic programming. Functionally, HAGLROS-conditioned macrophages promote EMT, angiogenesis, tumor-cell proliferation, and migratory behavior, highlighting how tumor-derived exosomal lncRNAs induce sustained transcriptional and signaling reprogramming rather than transient cytokine-mediated effects alone [32].

### MALAT1/POSTN/Hippo-YAP pathway in TNBC TAM reprogramming

Triple-negative breast cancer (TNBC)-derived exosomes enriched in metastasis-associated lung adenocarcinoma transcript 1 (MALAT1) can be internalized by macrophages, where they modulate stromal-remodeling and immunoregulatory signaling pathways. Mechanistically, exosomal MALAT1 upregulates periostin (POSTN) expression and promotes Hippo/YAP-associated signaling through increased nuclear localization and transcriptional activity of YAP. This signaling axis is associated with macrophage phenotypes characterized by tissue-remodeling, immune suppression, and tumor-supportive functions rather than strictly binary M1/M2 polarization states. Experimental inhibition of Hippo/YAP signaling attenuates MALAT1-mediated induction of immunoregulatory macrophage markers, supporting a functional role for this pathway in TNBC-associated immune remodeling. Functionally, MALAT1-conditioned macrophages enhance TNBC proliferation, invasion, and metastatic progression, indicating that tumor-derived EV-associated MALAT1 contributes to reciprocal tumor–stromal communication that facilitates dissemination and immune tolerance within the TME [25, 33, 34].

### m6A-Dependent LINC00657/miR-92b-3p/TGF-β bidirectional loop

Exosomal long intergenic non-coding RNA 00657 (LINC00657) represents a mechanistically important example of epitranscriptomically regulated tumor-derived lncRNAs involved in breast cancer immune remodeling. In breast cancer cells, LINC00657 undergoes increased m6A modification, which is associated with enhanced transcript stability and preferential incorporation into secreted exosomes. Following transfer into macrophages, exosomal LINC00657 functions as a ceRNA that sequesters microRNA-92b-3p (miR-92b-3p), thereby relieving repression of TGF-β-associated signaling components. Activation of TGF-β signaling promotes immunoregulatory macrophage polarization and enhances secretion of pro-tumorigenic mediators that subsequently reinforce breast cancer-cell proliferation, migration, and invasive capacity. This reciprocal signaling loop illustrates how epitranscriptomic regulation of lncRNAs can influence exosomal cargo selection and coordinate dynamic tumor–myeloid communication within the breast cancer microenvironment [18, 29, 35].

### LINC01943/ELAVL1-autophagy axis in radiotherapy resistance and macrophage reprogramming

Radiotherapy-exposed breast cancer cells release exosomes enriched in long intergenic non-protein coding RNA 1943 (LINC01943), which can subsequently reprogram macrophage phenotypes within the irradiated TME. Mechanistically, exosomal LINC01943 enhances embryonic lethal abnormal vision-like RNA-binding protein 1 (ELAVL1/HuR)-associated stabilization of autophagy-related transcripts, thereby increasing autophagic flux in recipient macrophages. This signaling program promotes macrophage phenotypes associated with immune suppression, tissue remodeling, and tumor support following radiotherapy exposure. These findings suggest that therapy-induced stress may reshape exosomal communication patterns that subsequently contribute to adaptive resistance and post-irradiation tumor survival [25, 36].

### SNHG16/miR-16-5p/SMAD5/CD73 axis in T-Cell immunosuppression

Breast cancer-derived exosomes also modulate non-macrophage immune compartments through transfer of lncRNA small nucleolar RNA host gene 16 (SNHG16). In this pathway, Vδ1 γδ T cells represent the principal immune recipient population rather than TAMs. Mechanistically, SNHG16 functions as a ceRNA that sequesters miR-16-5p, thereby derepressing SMAD family member 5 (SMAD5) and enhancing TGF-β1/SMAD5 signaling. This signaling cascade induces upregulation of CD73 (ecto-5′-nucleotidase), leading to increased extracellular adenosine production and subsequent suppression of T-cell proliferation and cytotoxic activity through A2A/A2B receptor signaling. Consequently, exosomal SNHG16 contributes to the establishment of an immunosuppressive microenvironment that promotes immune escape and tumor persistence through regulation of γδ T-cell function rather than direct macrophage-mediated effects [37].

### SNHG4/XPO5 in TNBC proliferation

Exosomal SNHG4 derived from TNBC cells has been reported to enhance proliferative and migratory phenotypes in recipient tumor cells through regulation of exportin-5 (XPO5), a critical mediator of precursor microRNA (pre-miRNA) nuclear export. Increased XPO5 expression may alter downstream miRNA maturation and transcriptomic regulation associated with cell-cycle progression and tumor-cell motility. Functional inhibition of XPO5 attenuates the pro-tumorigenic effects induced by SNHG4, supporting a potential role for SNHG4/XPO5-associated signaling in aggressive TNBC phenotypes. However, the precise downstream miRNA subsets and signaling networks responsible for these phenotypic alterations remain incompletely characterized. Importantly, this pathway primarily reflects tumor cell-autonomous exosomal signaling rather than direct macrophage-mediated communication [38].

### lnc-MTRNR2L12-3/Src/FAK in hypoxic angiogenesis

Hypoxic breast cancer cells secrete exosomes enriched in long non-coding RNA mitochondrial 2-like 12 − 3 (lnc-MTRNR2L12-3), which can be transferred to endothelial cells and promote angiogenic remodeling within the TME. Mechanistically, exosomal lnc-MTRNR2L12-3 enhances activation of the Src/focal adhesion kinase (FAK) signaling cascade, including increased phosphorylation of Src proto-oncogene tyrosine-protein kinase (Src) at tyrosine 416 (Tyr416) and FAK at tyrosine 397 (Tyr397). Downstream activation of cytoskeletal regulatory proteins, including paxillin (PXN) and Rho-associated coiled-coil-containing protein kinase (ROCK), facilitates endothelial migration, capillary-like tube formation, and vascular network assembly. These observations support the possibility that hypoxia-associated exosomal lncRNAs participate in angiogenic niche remodeling and metastatic adaptation through vascular signaling mechanisms that extend beyond canonical VEGF-associated pathways [39].

### NEAT1/miR-133b axis in paclitaxel resistance and motility

Exosomal nuclear-enriched abundant transcript 1 (NEAT1), released from paclitaxel-resistant breast cancer cells, can be internalized by drug-sensitive tumor-cell populations, where it promotes chemoresistance and enhanced migratory capacity. Mechanistically, NEAT1 functions as a ceRNA that suppresses microRNA-133b (miR-133b), thereby derepressing downstream pro-survival and pro-motility signaling pathways associated with cytoskeletal remodeling and stress adaptation. This exosome-mediated transfer facilitates horizontal dissemination of paclitaxel resistance among heterogeneous tumor-cell populations, reinforcing malignant plasticity and therapeutic failure through tumor–tumor communication rather than direct immune-cell modulation [40].

### HOTAIR-associated exosomal signaling in resistance-linked biomarker dynamics

Circulating exosomal HOX transcript antisense intergenic RNA (HOTAIR) has been detected in patients with breast cancer and is associated with aggressive clinicopathologic characteristics and unfavorable therapeutic outcomes. Although the precise recipient-cell signaling mechanisms of exosomal HOTAIR remain incompletely defined in clinical biomarker studies, elevated circulating levels are thought to reflect tumor-associated epigenetic activity and resistance-related transcriptional programs within the primary TME. Consequently, exosomal HOTAIR may serve as a minimally invasive biomarker for monitoring disease progression, therapeutic response, and resistance-associated tumor evolution in breast cancer [41, 42].

## Macrophage-derived exosomal lncRNAs reinforcing tumor progression

Although the majority of studies have focused on tumor-to-macrophage exosomal signaling, accumulating evidence indicates that TAMs also actively reshape malignant phenotypes through exosomal transfer of lncRNAs into breast cancer cells. These macrophage-derived exosomal lncRNAs regulate multiple processes associated with tumor progression, including metabolic adaptation, hypoxia signaling, epigenetic remodeling, EMT, and therapeutic resistance. Importantly, this reciprocal communication supports dynamic bidirectional signaling loops that reinforce tumor plasticity and tumor-supportive microenvironmental signaling programs. The pathways summarized in Table 2; Fig. 2 illustrate how macrophage-derived exosomal lncRNAs function as intercellular regulators of breast cancer progression through mechanistically distinct metabolic, metastatic, and epigenetic signaling programs.

**Table 2:** Functional roles of macrophage-derived exosomal lncRNAs in breast cancer

Macrophage exosomal lncRNA	Type of breast cancer	Macrophage phenotype/source	Experimental model	Primary molecular target(s)	Dominant signaling pathway(s) / functional axis	Functional impact on TME	References
**HISLA**	BC	TAM-derived extracellular vesicles	In vitro + in vivo + clinical correlation	HISLA binds PHD2, preventing hydroxylation-mediated degradation of HIF-1α	HISLA → PHD2 inhibition → enhanced HIF-1α signaling → sustained glycolytic gene transcription; lactate-driven feed-forward loop between TAMs and tumor cells	Enhances aerobic glycolysis, anti-apoptotic fitness, and chemoresistance; promotes metabolic co-evolution between TAMs and tumor cells	[19]
**MIR210HG**	TNBC	Hypoxic TAM-derived exosomes	In vitro + in vivo	MIR210HG increases HIF-1α protein levels and transcriptionally activates RASSF7 in TNBC cells	Hypoxic TAM exosomal MIR210HG → enhanced HIF-1α signaling → RASSF7 upregulation → EMT and metastatic reprogramming	Promotes TNBC migration, invasion, and vasculogenic mimicry under hypoxic TAM conditioning	[43]
**LINC00470**	BC	M2 macrophage-derived exosomes	In vitro + in vivo	LINC00470 upregulates MYC and DNMT3A; promotes promoter methylation and repression of miR-199a-3p	LINC00470 → MYC/DNMT3A activation → epigenetic silencing of miR-199a-3p → pro-tumor transcriptional reinforcement	Increases breast cancer cell proliferation, migration, and invasion after uptake of M2 macrophage exosomes	[44]
H19	BC	TAM-derived exosomes	Mechanistic evidence for TAM exosomal H19 in BC not yet established	Not defined	Putative Wnt/β-catenin, autophagy-associated signaling (limited TAM exosomal evidence)	Not yet supported by primary TAM exosome studies	[45]

C: Breast cancer; DNMT3A: DNA methyltransferase 3 alpha; EMT: Epithelial–mesenchymal transition; HIF-1α: Hypoxia-inducible factor 1 alpha; HISLA: HIF-1α-stabilizing long non-coding RNA; lncRNA: Long non-coding RNA; LINC00470: Long intergenic non-protein coding RNA 470; miR-199a-3p: MicroRNA-199a-3p; MIR210HG: MIR210 host gene; MYC: MYC proto-oncogene; PHD2: Prolyl hydroxylase domain-containing protein 2; RASSF7: Ras association domain family member 7; TAM: Tumor-associated macrophage; TNBC: Triple-negative breast cancer

### HISLA/PHD2/HIF-1α axis in metabolic symbiosis

TAM-derived EVs can transfer HISLA into breast cancer cells, where HISLA interferes with prolyl hydroxylase domain-containing protein 2 (PHD2)-mediated hydroxylation of HIF-1α. This interaction prevents proteasomal degradation of HIF-1α and sustains activation of glycolysis-associated transcriptional programs, thereby enhancing aerobic glycolysis, metabolic adaptation, and apoptotic resistance in recipient tumor cells. A defining feature of this pathway is its reciprocal metabolic reinforcement: lactate released by glycolytic tumor cells subsequently increases HISLA expression in macrophages, establishing a lactate-driven feed-forward communication loop between TAMs and breast cancer cells. This reciprocal immunometabolic signaling network contributes to chemotherapy resistance in vivo and has been associated with poor therapeutic response in patients with breast cancer, highlighting HISLA as one of the most mechanistically validated macrophage-derived exosomal lncRNA pathways currently described in breast cancer biology [19].

### MIR210HG/HIF-1α/RASSF7 in TNBC metastatic conditioning

Under hypoxic conditions, TAMs secrete exosomes enriched in microRNA-210 host gene (MIR210HG), which can be transferred into TNBC cells and promote aggressive metastatic phenotypes. Functional depletion of MIR210HG in hypoxic TAMs significantly attenuates exosome-mediated TNBC migration, invasion, and vasculogenic mimicry, supporting the biological relevance of this lncRNA in macrophage-driven tumor progression. Mechanistically, exosomal MIR210HG is associated with enhanced HIF-1α signaling and increased transcriptional activation of Ras association domain family member 7 (RASSF7), a pathway linked to EMT-associated transcriptional remodeling and metastatic adaptation. These findings indicate that hypoxic TAM-derived exosomal MIR210HG may contribute to metastatic adaptation in TNBC through coordinated regulation of hypoxia-responsive and EMT-associated signaling pathways [43].

### LINC00470/MYC/DNMT3A/miR-199a-3p in epigenetic silencing

Exosomes derived from M2-polarized macrophages can transfer long intergenic non-protein coding RNA 470 (LINC00470) into breast cancer cells, including MDA-MB-231 and MCF-7 models, where this lncRNA promotes epigenetic reprogramming associated with malignant progression. Following exosomal uptake, LINC00470 enhances expression of MYC proto-oncogene (MYC) and DNA methyltransferase 3 alpha (DNMT3A), leading to promoter hypermethylation and transcriptional repression of microRNA-199a-3p (miR-199a-3p). Reduced miR-199a-3p expression subsequently promotes tumor-cell proliferation, migration, and invasive behavior. Importantly, suppression of LINC00470 in M2 macrophage-derived exosomes reverses these oncogenic effects, supporting the existence of a macrophage-to-tumor epigenetic signaling axis that reinforces stable transcriptional and phenotypic reprogramming in breast cancer cells [44].

### H19/β-Catenin/Wnt in TAM-tumor feedback

Long non-coding RNA H19 (H19) is frequently overexpressed in breast cancer tissues and has been implicated in EMT, tumor progression, and resistance-associated signaling. Experimental studies have demonstrated that H19 modulates oncogenic pathways associated with Wnt/β-catenin signaling, where suppression of H19 reduces β-catenin activity, decreases EMT-related marker expression, and partially restores chemosensitivity in resistant breast cancer models. In addition, H19 has been identified in tumor-derived exosomes and implicated in intercellular communication associated with malignant progression and therapy resistance. However, despite increasing interest in H19-associated exosomal signaling, direct mechanistic evidence demonstrating macrophage-derived exosomal H19-mediated activation of Wnt/β-catenin signaling or autophagy-related pathways in recipient breast cancer cells remains limited. Therefore, although H19-associated exosomal signaling remains biologically plausible in the breast cancer microenvironment, definitive mechanistic evidence supporting a macrophage-to-tumor H19 signaling axis remains insufficient and requires further experimental validation [45].

## Therapeutic resistance, immune tolerance, and targeting the exosomal lncRNA network

The bidirectional exchange of exosomal lncRNAs between breast cancer cells and TAMs has direct implications for therapeutic resistance, immune tolerance, and disease persistence. Rather than acting as isolated molecular events, these EV-mediated signals connect metabolic adaptation, cytokine production, hypoxia-responsive signaling, epigenetic remodeling, and immune-cell dysfunction within the TME. As a result, resistance to chemotherapy, radiotherapy, targeted agents, and immune checkpoint blockade may arise not only from tumor cell–intrinsic alterations but also from adaptive communication between malignant cells and immune compartments [1, 19, 46].

A central example is macrophage-derived HISLA, which is transferred from TAM-derived EVs into breast cancer cells. HISLA interferes with PHD2-mediated hydroxylation and degradation of HIF-1α, thereby sustaining HIF-1α-dependent glycolytic transcriptional programs and reducing tumor-cell susceptibility to apoptosis under cytotoxic stress [19]. The resulting lactate-enriched microenvironment further enhances HISLA expression in macrophages, creating a reciprocal metabolic reinforcement loop that links TAM activity to glycolysis, apoptotic resistance, and chemoresistance. This pathway provides one of the strongest mechanistic examples of how macrophage-derived exosomal lncRNAs can convert metabolic stress into persistent therapeutic adaptation in breast cancer.

Tumor-derived exosomal lncRNAs also contribute to resistance by reshaping macrophage signaling programs that suppress anti-tumor immunity. Pathways involving HAGLROS/STAT3, MALAT1/Hippo-YAP, LINC00657/TGF-β, and radiotherapy-induced LINC01943/ELAVL1-associated autophagy promote macrophage phenotypes characterized by immunoregulatory cytokine production, tissue remodeling, angiogenesis, and tumor support [29, 32–36]. These macrophage states can increase IL-10, TGF-β, hypoxia-associated signaling, and extracellular matrix remodeling, thereby limiting cytotoxic lymphocyte infiltration and weakening immune-mediated tumor clearance. In this context, exosomal lncRNAs act as upstream modulators of the immune landscape rather than merely downstream markers of tumor progression.

Immune tolerance is further reinforced by exosomal lncRNA effects on immune populations beyond macrophages. Breast cancer-derived exosomal SNHG16 regulates Vδ1 γδ T cells through the miR-16-5p/SMAD5/TGF-β1 axis, leading to CD73 upregulation and increased extracellular adenosine production [37]. Adenosine signaling suppresses effector T-cell activation through A2A/A2B receptor pathways and may contribute to functional exhaustion and limited responsiveness to programmed cell death protein 1 (PD-1) or programmed death ligand 1 (PD-L1) blockade. Therefore, exosomal lncRNA-mediated immune escape should be interpreted as a multicellular process involving TAMs, γδ T cells, metabolic competition, cytokine signaling, and suppressive metabolite accumulation [37].

Epigenetic regulation provides another layer of resistance-associated communication. M2 macrophage-derived exosomal LINC00470 enhances MYC and DNMT3A expression in breast cancer cells, promoting promoter hypermethylation-associated repression of miR-199a-3p and supporting proliferation, invasion, and transcriptional persistence of malignant phenotypes [44]. Similarly, hypoxic TAM-derived exosomal MIR210HG has been linked to enhanced HIF-1α-responsive signaling, RASSF7 expression, EMT-associated remodeling, invasion, and vasculogenic mimicry in TNBC models [43]. These findings suggest that macrophage-derived exosomal lncRNAs can reinforce tumor-cell plasticity through metabolic, hypoxic, and epigenetic mechanisms, although pathway interpretation should remain limited to experimentally validated contexts.

Therapeutically, this biology supports strategies aimed at disrupting EV-mediated communication or selectively neutralizing oncogenic lncRNA cargo. Pharmacologic inhibition of neutral sphingomyelinase 2 using GW4869 can reduce exosome secretion and limit intercellular RNA transfer in experimental models, whereas suppression of Rab27a/Rab27b impairs multivesicular body docking and vesicle release [27, 47, 48]. More selective approaches include antisense oligonucleotides (ASOs) or small interfering RNAs (siRNAs) designed to target high-leverage lncRNAs such as HISLA, HAGLROS, LINC00657, or LINC01943. These RNA-directed strategies may weaken reciprocal tumor–macrophage resistance signaling without requiring global inhibition of EV biology, although delivery specificity, tissue penetration, off-target effects, and immune safety remain major translational barriers [19, 29, 32, 36, 49].

Macrophage-derived EVs may also be repurposed as therapeutic delivery platforms because of their natural interaction with tumor and immune compartments. Engineered macrophage exosomes have been explored as carriers for siRNAs, chemotherapeutic agents, and immune-stimulatory molecules, offering a potential approach to redirect macrophage–tumor communication toward anti-tumor activity [46]. However, clinical translation will require rigorous characterization of EV heterogeneity, quantitative assessment of lncRNA cargo abundance, validation of recipient-cell uptake, and integration of spatial transcriptomics, single-cell sequencing, and functional in vivo modeling to identify which exosomal lncRNA pathways are truly rate-limiting for resistance and immune escape.

## Integrative perspective: evidence strength, biological interpretation, and Ttranslational direction

The evidence summarized in this review supports the concept that exosomal lncRNAs are important regulators of reciprocal communication between breast cancer cells, TAMs, and additional immune or stromal compartments. However, the strength of evidence differs across pathways. HISLA represents a highly validated macrophage-to-tumor metabolic axis, whereas HAGLROS, MALAT1, LINC00657, LINC01943, MIR210HG, and LINC00470 provide pathway-specific evidence for macrophage polarization, hypoxia adaptation, radiotherapy resistance, metastatic remodeling, or epigenetic reprogramming [19, 29, 32–36, 43, 44]. By contrast, pathways involving SNHG16, SNHG4, NEAT1, lnc-MTRNR2L12-3, and HOTAIR should be interpreted as part of the broader breast cancer exosomal communication network because their principal recipient cells include γδ T cells, tumor cells, endothelial cells, or clinical biomarker compartments rather than TAMs alone [37–42].

This distinction is important for reviewer-level interpretation. Exosomal lncRNA signaling should not be presented as a single macrophage-restricted pathway or as universal convergence on one downstream cascade. Instead, current evidence supports a more realistic model in which tumor-derived and macrophage-derived exosomal lncRNAs regulate partially overlapping but mechanistically distinct signaling modules, including STAT3-associated macrophage activation, Hippo/YAP signaling, TGF-β-mediated immune remodeling, HIF-1α-dependent metabolic adaptation, DNMT3A-associated epigenetic regulation, CD73-mediated adenosine signaling, and Src/FAK-dependent angiogenic remodeling. Such a framework preserves the mechanistic diversity of the field while avoiding overgeneralization.

Several unresolved issues remain central to future progress. First, EV isolation methods and nomenclature differ across studies, making direct comparison difficult. Second, the abundance of specific lncRNAs within vesicles and their functional stoichiometry in recipient cells remain incompletely defined. Third, many mechanistic studies rely on overexpression, knockdown, or in vitro co-culture systems that may not fully reproduce endogenous EV transfer within human tumors. Finally, recipient-cell specificity is often insufficiently characterized, which can lead to overinterpretation of macrophage-dependent mechanisms. Addressing these limitations will require standardized EV characterization, quantitative RNA profiling, lineage-resolved uptake assays, spatial transcriptomics, and in vivo perturbation models.

Overall, exosomal lncRNAs should be viewed as dynamic mediators of tumor–immune adaptation rather than isolated biomarkers or single-pathway drivers. Their translational value will depend on identifying which lncRNA-containing EV signals are causally required for tumor progression, immune escape, and therapeutic resistance in vivo. A clinically useful framework will therefore need to distinguish functional driver cargo from passive vesicular passengers and determine whether targeting selected lncRNA nodes can disrupt resistance-associated communication without impairing protective immune responses.

## Conclusion

Breast cancer progression is shaped by reciprocal communication between malignant cells and the TME, where exosomal lncRNAs contribute to the regulation of macrophage polarization, immune suppression, metabolic adaptation, angiogenesis, EMT, epigenetic remodeling, and therapeutic resistance. The studies discussed in this review support a model in which tumor-derived and macrophage-derived exosomal lncRNAs coordinate multicellular signaling interactions rather than acting through one universal pathway. Mechanistically, validated pathways such as HISLA/HIF-1α, HAGLROS/STAT3, MALAT1/Hippo-YAP, LINC00657/TGF-β, MIR210HG/RASSF7, and LINC00470/MYC/DNMT3A illustrate how EV-mediated RNA transfer can reinforce tumor-supportive phenotypes in both macrophages and cancer cells. At the same time, exosomal lncRNAs also influence non-macrophage compartments, including γδ T cells, endothelial cells, and tumor-cell subpopulations, indicating that macrophage–tumor co-evolution occurs within a broader multicellular communication network.

Clinically, circulating exosomal lncRNAs such as HOTAIR, H19, MALAT1, and HISLA may serve as minimally invasive biomarkers of tumor progression, immune remodeling, and therapeutic response. Therapeutic strategies targeting EV biogenesis, vesicle trafficking, uptake mechanisms, or oncogenic lncRNA cargo offer promising but still experimental opportunities to disrupt resistance-associated signaling. Future studies should prioritize quantitative EV profiling, recipient-cell-specific validation, spatial and single-cell approaches, and in vivo functional testing to determine which exosomal lncRNA pathways are most actionable in breast cancer. In summary, exosomal lncRNAs represent important regulators of breast cancer tumor–immune communication and provide a biologically plausible framework for understanding therapy resistance, immune tolerance, and metastatic progression. Their greatest translational promise lies not in treating them as isolated molecules, but in defining how they participate in coordinated intercellular signaling networks that can be measured, disrupted, or therapeutically redirected.

## Data Availability

No datasets were generated or analysed during the current study.

## Declarations

### Declaration on the Use of Generative AI in Scientific Writing

In the preparation of this manuscript, generative artificial intelligence tools were utilized exclusively to improve linguistic clarity and grammatical precision. All generated content was carefully reviewed, critically revised as needed, and formally approved by the authors, who retain full responsibility for the accuracy, integrity, and final version of the work.
